# Softer More Frequent Stools in Infants With Difficult Stooling Fed Hydrolyzed Protein Formula With Added Prebiotics: Randomized Controlled Trial

**DOI:** 10.3389/fped.2022.894626

**Published:** 2022-05-31

**Authors:** Veronica Fabrizio, Cheryl L. Harris, Kelly R. Walsh, Jennifer L. Wampler, Weihong Zhuang, Steven S. Wu

**Affiliations:** ^1^Medical and Scientific Affairs, Reckitt | Mead Johnson Nutrition Institute (MJNI), Evansville, IN, United States; ^2^Department of Pediatrics, Indiana University School of Medicine, Indianapolis, IN, United States

**Keywords:** infant feeding, infant formula, tolerance, polydextrose, galactooligosaccharides (GOS)

## Abstract

**Objective:**

To evaluate stool consistency in infants with reported hard or infrequent stools fed hydrolyzed protein formula with added prebiotics designed to promote stool softening.

**Methods:**

In this multi-center, double-blind, controlled study, eligible infants (28–300 days of age at enrollment) were randomized to: partially hydrolyzed cow’s milk protein formula (PHF, 75% carbohydrate as lactose; 12 mg Mg/100 kcal; *n* = 49) or routine intact protein cow’s milk-based infant formula (Control, 92% carbohydrate as lactose; 8 mg Mg/100 kcal; *n* = 51) over a 14-day period. Both formulas had a prebiotic blend (polydextrose and galactooligosaccharides, 4 g/L; 1:1 ratio). Parent-reported stool consistency (hard = 1 through watery = 5) and other daily outcomes were collected by diary. Endpoint stool consistency (mean score over last 3 days of study feeding) was the primary outcome. Adverse events were recorded.

**Results:**

Baseline stool consistency (Control: 1.4 ± 0.1, PHF: 1.4 ± 0.1) and frequency were similar between groups; the majority had hard (*n* = 61, 64%) or formed (*n* = 30, 32%) stools. Stool consistency became softer over Day 1–3 (Control: 2.5 ± 0.1, PHF: 2.6 ± 0.1) and remained similar from Day 4 to 6 through study end (*post hoc* analysis). For PHF vs Control, endpoint stool consistency was significantly softer (3.4 ± 0.1 vs 3.0 ± 0.1; *P* = 0.019) and frequency significantly higher (1.5 ± 0.1 vs 1.0 ± 0.1; *P* = 0.002). Crying, fussing, and appearance of pain during stooling decreased from baseline to study end in both groups. Formula intake, infant fussiness and incidence of adverse events were similar between groups.

**Conclusion:**

An infant formula designed to promote stool softening was well-tolerated and associated with softer, more frequent stools in infants with reported hard or infrequent stools.

## Introduction

Parental complaint of difficult stooling in infants is common; however, data related to the incidence are limited, largely because assessment is subjective. Prevalence of constipation has been reported between 8 and 18% (1, 2), though parental perception of difficult stooling in infants is likely higher. Changes in infant stooling patterns often accompany feeding transitions, such as changes in source of milk and/or introduction of solid foods. Constipation is a source of discomfort for infants as well as anxiety and concern for parents (1, 3) and hard stool is more prevalent in infants who are not exclusively breastfed (2, 4). Therefore, nutritional adaptations of formulas to mitigate difficult stooling in infants are warranted.

Whey and casein proteins are present in human milk and in bovine milk-based infant formulas in various ratios. Dietary protein composition can influence digestion and gastrointestinal (GI) motility, due in part to structural differences between intact casein and whey proteins and subsequent behavior in the acidic milieu of the stomach. Hydrolysis of proteins used in infant formulas may also impact GI motility. For example, the percentage of gastric residual activity was significantly lower in infants fed whey hydrolysate formula versus intact protein formulas (5). Gastric motility changes may ultimately alter stooling patterns. Increased gastric transit time (6) and passage of significantly more stool have been demonstrated in preterm infants fed hydrolyzed protein (7). Higher stool frequency and more watery stools in infants fed extensively hydrolyzed versus intact protein formula (8) and stool consistency differences in infants fed partially hydrolyzed formula versus intact soy formula (9) have been demonstrated. Softer stools have also been reported in infants fed partially hydrolyzed protein formulas (10–12). Partial hydrolysis of protein is one potential nutritional strategy for softening stools.

Other components that could affect stool patterns include human milk oligosaccharides (HMOs), magnesium (Mg), and lactose. HMOs are the third most abundant macro component of human milk and have properties that promote infant health (13) and prebiotic oligosaccharides are used in some commercially available infant formulas to emulate HMO functionality. For example, previous studies have demonstrated softer stools in healthy term infants fed routine infant formula with an added prebiotic blend of polydextrose (PDX) and galactooligosaccharides (GOS) versus no added prebiotics (14–17). These studies, however, did not assess infants with perceived hard stools or uncomfortable stooling. Dietary Mg can promote stool softening via colonic osmotic effects or stimulation of cholecystokinin release and concomitant intestinal fluid and electrolyte accumulation (18). In infants receiving standard infant formula, stool water content increased and parental perception of constipation improved in infants after receiving formula with higher lactose and Mg for 2 weeks (3); similar findings were reported in a study of higher Mg formula alone (4). Lactose is the primary carbohydrate in human milk, representing 80% of total carbohydrate (13, 19). Dietary lactose has been known to cause osmotic properties of luminal retention or secretion of water when not completely digested and absorbed in the gut (3, 20).

Consequently, these nutritional adaptations in infant formula are potential dietary strategies to support normal infant stool patterns. The aim of the current study was to investigate the effects on stool characteristics of a novel formula designed for infants with parental report of hard or infrequent stools that had partially hydrolyzed cow’s milk protein, prebiotics, higher magnesium, and lactose as the predominate source of carbohydrate.

## Materials and Methods

### Study Population

Infants were recruited at 10 clinical sites in the United States. Eligible infants were: 28–300 days of age at randomization, were not currently diagnosed with cow’s milk allergy, and met key criteria for difficult stooling within the 10-day period prior to randomization: had a history of parent-reported stool consistency (scaled as hard = 1, formed = 2, mushy = 3, unformed or seedy = 4, watery = 5) of (1) at least two hard stools or (2) two or more hard or formed stools and 48 consecutive hours without a bowel movement. Full inclusion and exclusion criteria are provided in Table 1.

**TABLE 1:** Participant inclusion and exclusion criteria.

Inclusion criteria	Exclusion criteria
• 28–300 days of age at randomization, inclusive (day of birth is considered day 0) • Singleton birth • Gestational age of ł35 weeks (34 weeks and six days is considered 34 weeks gestational age) • Received a minimum of 18 fl oz of infant formula in the 24 h prior to randomization • History of: • At least two Grade 1 stools (using 5-point stool consistency scale where 1 = hard) over the last 10-day period OR • Two or more stools of a minimum Grade 2 consistency (using 5-point stool consistency scale where 2 = formed) AND 48 consecutive hours without a bowel movement over the last 10-day period • Parent or legal guardian who will be attending study visits and completing diaries can read, understand, and speak English •Parent or legal guardian agrees not to enroll infant in another interventional clinical study while participating in this study • Signed informed consent obtained from parent or legal guardian, for infant’s participation in the study and, if the infant will receive mother’s-own breast milk during the study, the mother has signed consent • Signed authorization obtained from parent or legal guardian, to use and/or disclose protected health information for infant from birth through the length of the study period and, if the infant will receive mother’s-own breast milk during the study, the mother has signed the authorization	• Current diagnosis of cow’s milk protein allergy or intolerance • Parent or legal guardian agrees to make an effort to avoid use of any oral product intended to soften the stool and enemas of any type for the infant during the study period • Any abdominal or gastrointestinal surgery prior to randomization • Any use of anesthesia or opioid medication within the 10 days prior to randomization • Use of oral, intramuscular or intravenous antibiotics within the 7 days prior to randomization • Use of any oral product intended to soften the stool or enemas of any type within 72 h prior to randomization • Acute gastrointestinal illness in the 48 h prior to randomization • Use of extensively hydrolyzed or amino acid formula at randomization •Organic cause of constipation, such as Hirschsprung’s disease, spina bifida, hypothyroidism or other metabolic abnormalities, renal abnormalities, Down syndrome or other significant developmental disorders • History of underlying metabolic or chronic disease; congenital malformation; or any other condition which, in the opinion of the Investigator, is likely to interfere with: the ability of the infant to ingest food, the normal growth and development of the infant, or the evaluation of the infant • Maternal use of opioid medication within the 10 days prior to randomization if the infant is receiving mother’s-own breast milk at randomization

### Study Design

In this multicenter, double-blind, randomized, controlled, parallel-group, prospective trial, parents or guardians provided written informed consent prior to enrollment. The research protocol and informed consent form observing the Declaration of Helsinki (including October 1996 amendment) were approved by Shulman IRB (now known as Advarra, Columbia, MD, United States) on May 30, 2017. The study complied with good clinical practices. Participants were enrolled starting August 24, 2017 through October 30, 2018. Participants were randomly assigned to receive one of two study formulas (Mead Johnson Nutrition, Evansville, IN, United States; Table 2): a routine cow’s milk-based infant formula (Control, marketed Enfamil^®^; 92% carbohydrate as lactose, 8 mg Mg/100 kcal) or a partially hydrolyzed cow’s milk protein (∼70% whey) formula designed for perceived stooling issues (PHF; 75% carbohydrate as lactose, 12 mg Mg/100 kcal) over a 14-day feeding period. Both formulas had an added prebiotic blend (PDX and GOS, 1:1 ratio; 4 g/L) (PDX: Litesse^®^ Two Polydextrose, Danisco; GOS: Vivinal^®^ GOS Galactooligosaccharide; Friesland Foods Domo) and an added bovine milk fat globule membrane (bMFGM) ingredient (5 g/L, whey protein-lipid concentrate; Lacprodan^®^ MFGM-10, Arla Foods Ingredients P/S, Denmark).

**TABLE 2:** Nutrient composition per 100 kcal.

Nutrient	Study formula, target values	
	Control	PHF
Total protein, g^a,b^	2	2.3
Total Fat, g	5.3	5.3
Linoleic acid, mg	800	780
α-Linolenic acid, mg	75	72
ARA, mg	34	34
DHA, mg	17	17
Total carbohydrate, g^c,d^	11.3	11.1
Vitamin A, IU	300	300
Vitamin D, IU	60	60
Vitamin E, IU	2	2
Vitamin K, mcg	9	9
Thiamin, mcg	80	80
Riboflavin, mcg	140	140
Vitamin B6, mcg	60	60
Vitamin B12, mcg	0.3	0.3
Niacin, mcg	1000	1000
Folic acid, mcg	16	16
Pantothenic acid, mcg	500	500
Biotin, mcg	3	3
Vitamin C, mg	12	12
Choline, mg	24	24
Inositol, mg	24	24
Calcium, mg	78	82
Phosphorus, mg	43	46
Magnesium, mg	8	12
Iron, mg	1.8	1.5
Zinc, mg	1	1
Manganese, mcg	15	15
Copper, mcg	75	75
Iodine, mcg	15	15
Selenium, mcg	2.8	2.8
Sodium, mg	27	36
Potassium, mg	108	108
Chloride, mg	63	63

*^a^Control: intact cow’s milk protein; Investigational: partially hydrolyzed cow’s milk protein.*

*^b^Protein source has added bovine milk fat globule membrane (bMFGM) ingredient (5 g/L, whey protein-lipid concentrate).*

*^c^Control and PHF each have prebiotic oligosaccharides (0.6 g; prebiotic blend of PDX and GOS, 1:1 ratio; 4 g/L).*

*^d^For Control, Lactose: ∼92% of total carbohydrates; For PHF, Lactose: ∼75% of total carbohydrates.*

### Randomization and Study Group Allocation

The study sponsor created computer-generated randomization schedules provided in sealed envelopes for each study site that were stratified by age: (1) 28–180 days of age or (2) >180–300 days of age. Study formula was assigned by opening the next sequential envelope at the study site. Study formulas, each designated by two unique codes known only to the sponsor, were dispensed to parents at randomization. Neither the product labels nor the sealed envelopes allowed direct unblinding by the study site. Personnel responsible for monitoring the study were also blinded to study product identification. Blinding for a participant could be broken by study sponsor in the event of a medical emergency in which knowledge of the study formula was critical to the participant’s management. In this study, it was not necessary to break the study code prematurely.

### Study Outcomes

Study visits corresponded to randomization (28–300 days of age; baseline) and study visit 2 (14–19 days after starting study formula) or early study exit. Parental 3-day recall of stool consistency seen most often, the approximate number of bowel movements per day, and how fussy the infant had been and behavior during stooling, in addition to the frequency of probiotic use during the week prior to the study visit were collected at baseline. Participants could receive feedings of breast milk in addition to study formula during the study. Parents were instructed to start study formula feeding with the next feeding following the baseline study visit and to start daily diary recording. The daily diary was used to collect a 24-h recall of formula intake, stool characteristics (stool frequency and consistency), fussiness, behavior during bowel movements (crying, fussing, or appearing to be in pain while passing or attempting to pass stool), and use of any stool softening measures other than the study formula. Responses for amount of fussiness were scaled (not fussy = 0, slightly fussy = 1, moderately fussy = 2, very fussy = 3, extremely fussy = 4). Parents were asked to complete the daily diary at approximately the same time each evening. Breastfeeding mothers’ use of antibiotics or opioid medication and participant use of antibiotics, probiotics, and opioid medication were recorded throughout the study period. Medically confirmed adverse events were collected throughout the study period and coded according to specific event (e.g., otitis media, colic, etc.) and the body system involved. At study end, 3-day parental recall of stool consistency, how fussy the infant had been, and behavior during stooling was recorded, in addition to a recall of changes in spitting up and/or vomiting and gassiness relative to normal over the course of the study.

### Statistical Analysis

Endpoint stool consistency (mean score) was the primary outcome. The endpoint period was defined as the last 3 days of study formula consumption prior to infant use of an enema or oral laxative or use of an opioid medication by the breastfeeding mother or infant. If no stool consistency data was available (1) from the daily diary during the endpoint period, the stool consistency score from the study end questionnaire was used as the endpoint score or (2) from daily diaries or the study end questionnaire, the baseline score was used as the endpoint score. A sample size of 50 participants per feeding group with no adjustments for dropouts was estimated to allow detection of a group difference of 0.4 (SD = 0.7) in endpoint stool consistency scores (α = 0.05, two-tailed test, 80% power). Endpoint stool consistency was analyzed by ANOVA including terms for study group and age category (≤180 days, >180 days at baseline). A *post hoc* analysis by ANOVA was performed to compare stool consistency (mean score) for the first three diary days. Stool consistency scores were also averaged over 3-day intervals and plotted.

Secondary outcome measures included stool frequency and other tolerance measures, study formula intake, and medically confirmed adverse events. Birth anthropometrics, age at baseline and body weight (z-scores) at baseline were analyzed by ANOVA including a term for study group. Endpoint stool frequency and fussiness were analyzed by ANOVA including terms for study group and age category. Changes in spitting up/vomiting level and gassiness relative to normal at study end were analyzed using the Cochran-Mantel-Haenszel (CMH) row mean score test. Participant characteristics (race, ethnicity, sex); the number of participants with discomfort symptoms during or while attempting to stool (crying, fussing, appearance of pain) during the endpoint period; the number of participants who consumed at least 18 oz of study formula per day on at least 80% of the days in the analysis period; and the incidence of adverse events was analyzed using Fisher’s exact test. All tests were conducted at α = 0.05. All analyses were conducted using SAS version 9.4 (Cary, NC, United States).

## Results

### Participants

**FIGURE 1:**
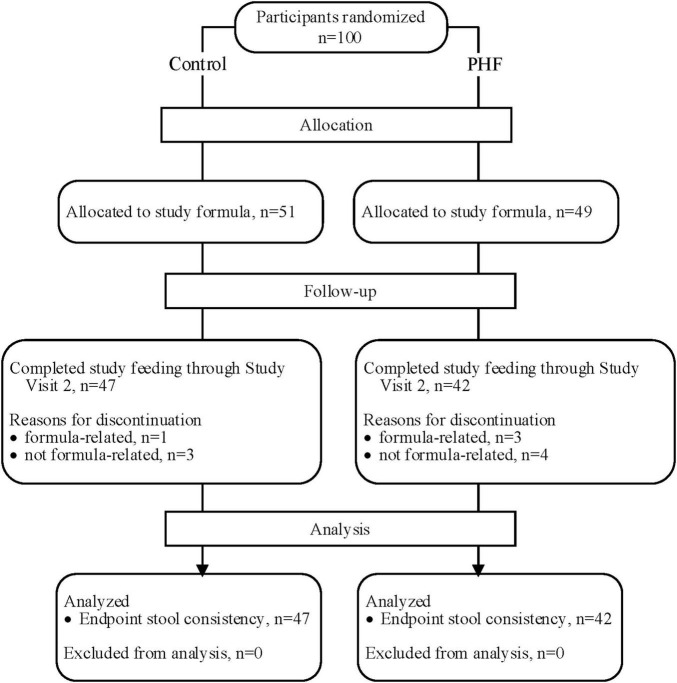
Flow of study participants.

A total of 100 participants were enrolled and randomized (Control: 51; PHF: 49) and 89 infants completed study feeding up to study visit 2 (Control: 47; PHF: 42) (Figure 1). Birth anthropometric measures, as well as sex, race, and ethnic distribution were similar between groups and no group differences in age or weight z-score were detected at study enrollment (Table 3).

**TABLE 3:** Infant characteristics at birth and study baseline.

Infant characteristic	Study group		*P*
	Control	PHF	
Sex, *n* (%)			1.000
Female	24 (47)	23 (47)	
Male	27 (53)	26 (53)	
Race, *n* (%)			0.690
White	42 (82)	40 (82)	
Black	5 (10)	3 (6)	
Other	4 (8)	6 (12)	
Ethnicity, *n* (%)			0.071
Hispanic	10 (20)	3 (6)	
Not Hispanic	40 (80)	46 (94)	
Birth anthropometrics, mean ± SE			
Weight, g	3281.2 ± 64.3	3267.6 ± 65.6	0.883
Length, cm	50.4 ± 0.3	50.3 ± 0.3	0.792
Head circumference, cm	34.1 ± 0.2	34.0 ± 0.2	0.781
Measures at baseline, mean ± SE			
Weight, z-score	−0.2 ± 0.1	−0.4 ± 0.1	0.236
Age (days)	110.7 ± 10.1	109.5 ± 10.3	0.933

### Baseline and Endpoint Stool Consistency and Frequency

**FIGURE 2:**
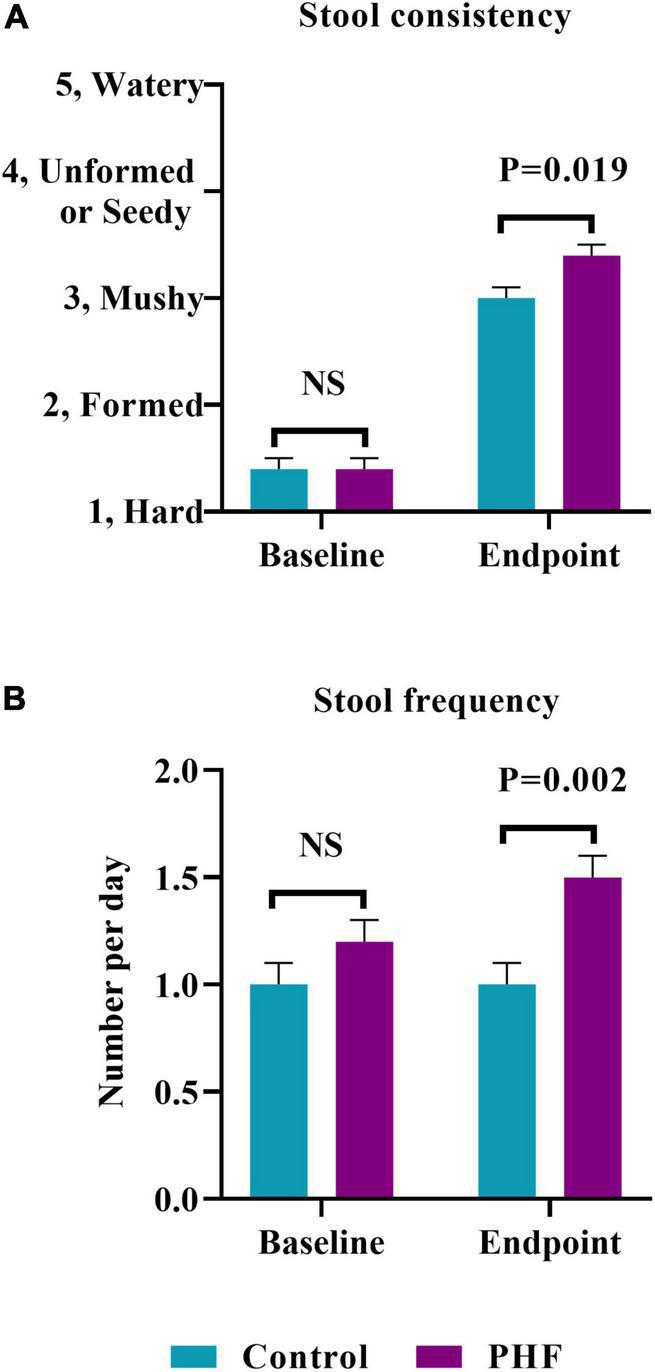
**(A)** Stool consistency (mean ± SE) at Study Baseline and Endpoint. Parent-reported stool consistency was scaled as: hard, 1; formed, 2; mushy, 3; unformed or seedy, 4; watery and **(B)** stool frequency (mean ± SE) at Baseline and Endpoint which was parent-reported as number/day.

Baseline stool consistency (mean ± SE) was not significantly different between groups (Control: 1.4 ± 0.1, PHF: 1.4 ± 0.1; Figure 2A). The highest proportions in both groups reported the consistency as “hard” (Control: *n* = 30, 63%, PHF: *n* = 31, 66%) followed by “formed” (Control: *n* = 16, 33%, PHF: *n* = 14, 30%). Endpoint stool consistency was significantly softer in the PHF (3.4 ± 0.1) compared to the Control (3.0 ± 0.1, *P* = 0.019; Figure 2A). Baseline stool frequency (mean ± SE) was not significantly different between groups (Control: 1.0 ± 0.1, PHF: 1.2 ± 0.1; Figure 2B). Endpoint stool frequency was significantly different between groups, with a higher frequency reported in the PHF group (Control: 1.0 ± 0.1, PHF: 1.5 ± 0.1; *P* = 0.002; Figure 2B).

### Stool Consistency Over Time

**FIGURE 3:**
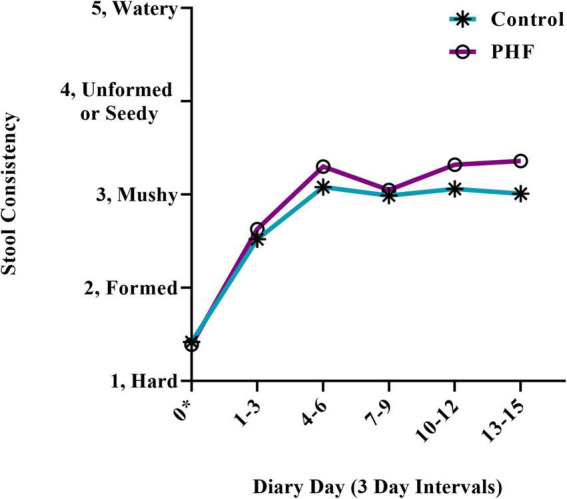
Stool consistency scores over time. Means (±SD) for parent-reported stool consistency for 3-day diary intervals were plotted. Stool consistency scores for the first 3 diary days were compared by *post hoc* analysis. *For Day 0 (Baseline), scores refer to the 3 days prior to Study Visit 1.

By *post hoc* analysis, stool consistency over time (Figure 3) became softer in both groups over Days 1–3 with no significant difference between groups (Control: 2.5 ± 0.1, PHF: 2.6 ± 0.1). Plotted means remained similar from Days 1–3 to Days 4–6 and through Days 13–15.

### Digestive Symptom Assessment

**FIGURE 4:**
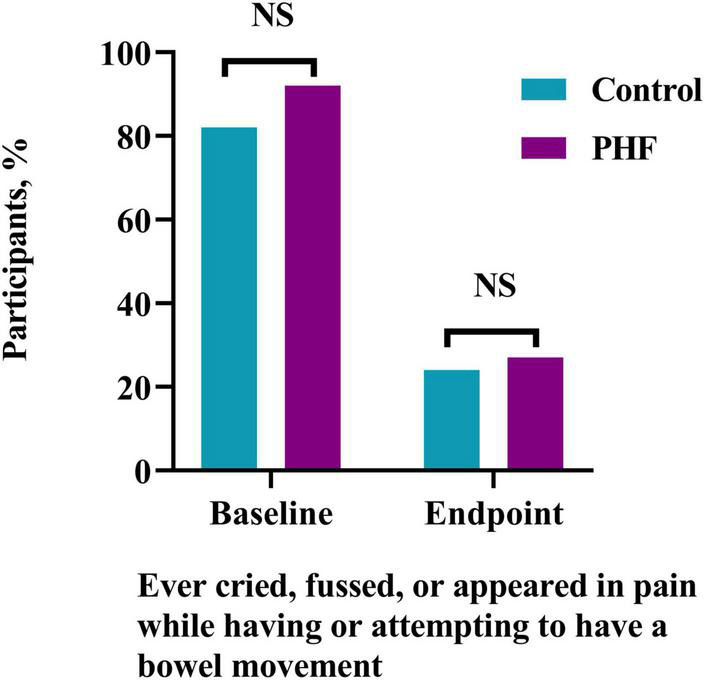
Participants (%) who ever cried, fussed or appeared in pain while having or attempting to have a bowel movement at Study Baseline and Endpoint.

Baseline (Control: 42, 82%: PHF: 45, 92%) and endpoint (Control: 12, 24%: PHF: 13, 27%) discomfort symptoms during or while attempting to stool (crying, fussing, and appearance of pain) were not significantly different between groups (Figure 4). Endpoint symptoms were low in both groups. In addition, fussiness at baseline (Control: 1.8 ± 0.2, PHF: 1.9 ± 0.2) and endpoint (Control: 0.8 ± 0.2, PHF: 0.9 ± 0.2) was not significantly different between study groups. Changes in spitting up/vomiting and gassiness relative to normal were similar between groups (Table 4).

**TABLE 4:** Changes in spitting up/vomiting and gassiness over the course of the study.

	Study group		*P*
	Control	PHF	
Change in occurrences of spitting up and/or vomiting, *n* (%)			0.476
Decreased	12 (24)	17 (36)	
Stayed the same	27 (53)	18 (38)	
Increased	12 (24)	12 (26)	
Gassiness Relative to Normal, *n* (%)			0.781
Less gas than normal	14 (27)	18 (38)	
About the same as normal	24 (47)	14 (30)	
More gas than normal	13 (25)	15 (32)	

### Study Feeding, Other Interventions, and Adverse Events

No significant differences were detected between study groups in duration of study feeding in days (Control: 15.3 ± 0.6, PHF: 14.8 ± 0.6), formula intake (participants who consumed at least 18 oz/day on at least 80% of study days, Control: *n* = 46, 92%, PHF: *n* = 43, 93%), or discontinuation of formula prior to study visit 2 for formula-related (Control: *n* = 1, 2%, PHF: *n* = 3, 6%) or non-formula related issues Control: *n* = 3, 6%, PHF: *n* = 4, 8%). Four infants (Control, *n* = 2; PHF, *n* = 2) received partial breastfeeding.

Data was collected on other common measures to promote infant stooling during the study. More than one measure may have been used per participant, but overall use remained low and included oral laxatives (Control: *n* = 1, PHF: *n* = 1), juice (Control: *n* = 1, PHF: *n* = 3), rectal stimulation or suppository (Control: *n* = 2, PHF: *n* = 2), probiotics (Control: *n* = 0, PHF: *n* = 1), other methods (Control: *n* = 3, PHF: *n* = 3), and method not specified (Control: *n* = 0, PHF: *n* = 1). No enema or opioid use was reported during the study. There were no significant differences detected between groups for number of participants with at least one antibiotic use (Control: *n* = 2, 4%, PHF: *n* = 5, 10%). There were no significant differences detected between groups in the number of participants who experienced at least one adverse event (Control: *n* = 8, 16%, PHF: *n* = 14, 29%).

## Discussion

Parental concern about infant stooling, and especially the perception of “constipation” (including symptoms of hard, infrequent, and/or difficult-to-pass stools) is one of the most reported GI problems in infancy. Though figures vary by definition used, prevalence has been reported from 8 to 18% within the first several months of life (1, 2) and is more common in infants receiving formula compared to human milk (2, 4). Therefore, previous ingredient modifications have been evaluated in past studies, with softer and/or more frequent stools reported in association with hydrolyzed protein (6, 7, 9–12), prebiotics (14–17), and higher concentrations of lactose (3) and magnesium (3, 4).

The incorporation of hydrolyzed protein in formula has been associated with favorable changes in stooling pattern in studies of both preterm (6, 7) and term (8–12) infants. For example, increased frequency has been observed in preterm (7) and term infants (11) receiving hydrolyzed protein formula and softer stools have been reported in infants receiving partially hydrolyzed protein formula relative to both soy (9) and intact cow milk-based formulas (10, 11). Altered gut motility likely contributes to these effects, as suggested by evidence for faster gastric emptying and increased GI transit in preterm (6, 7, 21) and term infants (5) receiving hydrolyzed protein formulas. One possible mechanism is that hydrolyzed protein increases the intraluminal osmotic load, causing higher motilin levels and decreasing the activity of milk protein-derived opioid receptor agonists (11). A further possibility is that the partial hydrolysis of cow milk proteins may potentially reduce allergenicity to a limited extent. Whereas it is widely recognized that formulas based on extensively hydrolyzed protein are the preferred choice for infants with known cow milk allergy, partial hydrolysis of cow milk protein may remove at least some of the sensitizing epitopes (22). Thus, in some infants, a partial hydrolyzate could potentially improve stooling symptoms by affecting low-grade or unrecognized cow milk allergy/sensitivity, since GI motility can be altered in response to inflammation and secretion of histamine and serotonin in allergic individuals (23, 24).

A prebiotic has been defined as: “a substrate that is selectively utilized by host microorganisms conferring a health benefit” (25). Prebiotic HMOs inhibit the binding of pathogens and are metabolized by gut bacteria in infants receiving human milk which promotes the growth of certain microorganisms (13, 26). Soluble fibers, such as PDX and GOS, are prebiotic oligosaccharides that share some functional characteristics and have been used in some formulas to mimic the microbiome-modulating functionality of HMOs. The prebiotic blend of PDX and GOS has been demonstrated to promote softer stools (14–17) and bifidobacterial abundance closer to human milk (14) in infants receiving formula with compared to without added prebiotics. In the current study, both had the same prebiotic blend of PDX and GOS which likely contributed to softening of stool over the course of the study for all infants but would not be expected to contribute to differences between groups.

The recognized osmotic laxative effect and increased motility of Mg is associated with softer stools (3, 18). Previously, a higher-magnesium formula vs routine formula (8.5 mg/100 mL Mg vs 5.1 mg/100 mL Mg) was associated with a significantly higher stool frequency, likelihood of achieving normal stooling parameters, and parental satisfaction in infants with functional constipation (4). The difference in Mg content between formulas in the previous study is comparable to the difference in Mg content in the current study formulas (PHF at 8 mg/100 mL Mg vs control at 5.3 mg/100 mL Mg).

In the current study, the control formula had a higher lactose content than the investigational PHF. However, lactose content for the PHF was higher than other similar partially hydrolyzed formulas. Whereas the majority of lactose should be digested and absorbed in the infant small intestine under normal conditions, a small fraction of undigested lactose may reach the colon where it can exert an osmotic effect (3). Therefore, higher lactose may also contribute maintenance of soft stooling (3). In this case, the investigational formula had lower lactose (75 vs 92%) compared to the control, intact protein formula, due to the high potential for Maillard reactions between lactose and exposed amine functional groups in the hydrolyzed protein (27, 28). Nevertheless, higher lactose in comparison to other hydrolyzate formulas (typically 50% of carbohydrate or less) may have played a minor role in addition to the other factors discussed.

In addition to stool consistency and frequency changes, the investigational formula was well-tolerated, with no significant group differences in: adverse events, study discontinuation, or duration of study feeding; need for additional interventions (such as rectal stimulation); parental report of overall fussiness; or behaviors suggestive of pain or discomfort during stooling (which improved significantly over the course of the study in both groups). Of note, there were no group differences in the reported incidence of diarrhea, indicating that the stool softening effects of the formula did not “overcorrect” and lead to excessive cathartic or laxative effect.

A study limitation was the inability to distinguish between specific effects of individual ingredients within the investigational formula matrix. However, previous studies have also evaluated multiple modifications in investigational formulas, such as formula that has partially hydrolyzed protein, prebiotic oligosaccharides and palmitic acid (29, 30) or formula that has higher lactose and Mg (3). In addition, although stool was significantly softer in PHF versus the control group, stool softened in both groups over time. In the current study, the prebiotic blend of PDX and GOS [associated with softer stools in prior studies (14–17)] and lactose in both formulas (3), natural development of GI function with age, and placebo effect could also contribute to stool softening. Though infant formula was the predominant feeding source, and neither formula feeding, partial breastfeeding, nor the rare use of fruit juice differed significantly between groups, additional differences in complementary feeds could have been present, though such group differences should be minimized due to randomization.

In conclusion the present study demonstrates that an infant formula that has partially hydrolyzed protein, a prebiotic blend, 75% carbohydrate as lactose, and increased Mg designed to promote stool softening was both well-tolerated and associated with softer, more frequent bowel movements.

## Data Availability Statement

The authors and study sponsor encourage and support the responsible and ethical sharing of data from clinical trials. De-identified participant data from the final research dataset used in the published manuscript may only be shared under the terms of a Data Use Agreement. Requests may be directed to the corresponding author. This study adheres to CONSORT guidelines.

## Ethics Statement

The studies involving human participants were reviewed and approved by Shulman IRB (now known as Advarra, Columbia, MD, United States). Written informed consent to participate in this study was provided by the participants’ parents or legal guardians.

## Author Contributions

SSW conceptualized and designed the study. CLH and WZ participated in study design and performed statistical analyses. KRW participated in study design and study formula design. JLW participated in study design. VF drafted the initial manuscript. All authors interpreted the data, contributed to the intellectual content, critically reviewed and revised the manuscript, accountable for all aspects of the work, and agreed to the final version of the manuscript.

## Conflict of Interest

VF, CLH, JLW, WZ, and SSW are currently employed by Reckitt|Mead Johnson Nutrition, Evansville, IN, United States. KRW was previously employed by Reckitt|Mead Johnson Nutrition, Evansville, IN, United States.

## Publisher’s Note

All claims expressed in this article are solely those of the authors and do not necessarily represent those of their affiliated organizations, or those of the publisher, the editors and the reviewers. Any product that may be evaluated in this article, or claim that may be made by its manufacturer, is not guaranteed or endorsed by the publisher.
